# Exploring the Mechanisms of Amino Acid and Bioactive Constituent Formation During Fruiting Body Development in *Lyophyllum decastes* by Metabolomic and Transcriptomic Analyses

**DOI:** 10.3390/jof11080586

**Published:** 2025-08-08

**Authors:** Lidan Liang, Peijin Zhang, Jiayan Lu, Wenjin Han, Pengfei Ren, Yufei Lan, Qingji Wang, Zhuang Li, Li Meng

**Affiliations:** 1 College of Plant Protection, Shandong Agricultural University, Tai’an 271018, China; lidanliang2023@163.com (L.L.); zhangpeijin0615@126.com (P.Z.); ljy19563365827@163.com (J.L.); hanwenjin2005@163.com (W.H.); 2 State Key Laboratory of Nutrient Use and Management, Shandong Academy of Agricultural Sciences, Jinan 250100, China; pengfei_jinan@163.com; 3 Tai’an Academy of Agricultural Sciences, Tai’an 271000, China; lanyufei526@163.com

**Keywords:** *Lyophyllum decastes*, flavor amino acid metabolism, polysaccharide biosynthesis, secondary metabolism, fruiting body development

## Abstract

*Lyophyllum decastes*, a common edible mushroom, is prized for its exceptional taste and rich nutritional composition. The concentrations of amino acids and polysaccharides in the fruiting body exhibited a dynamic increase throughout development, reaching their highest levels in the maturation stages, with values of 45,107.39 μg/g and 13.66 mg/g, respectively. Integrated metabolomic and transcriptomic analyses uncovered dynamic metabolites changing during the transition from vegetative growth to reproductive development. Several differentially expressed genes (DEGs) and differentially accumulated metabolites (DAMs) were identified, associated with secondary metabolite, amino acid, and carbohydrate metabolism. The shift in metabolites was linked to key nutrient synthesis, explaining the abundant production of amino acids and polysaccharides at maturity. Our results provide novel insights into the developmental biology of *L. decastes*, demonstrating that this mushroom is a valuable source of bioactive compounds and contributing to the optimization of cultivation strategies, as well as improving research into its application as a functional food and nutraceutical source.

## 1. Introduction

Mushrooms have been extensively used as functional foods in Asia for centuries, primarily due to their low-fat content, high protein levels, and diverse bioactive components [1]. *Lyophyllum decastes*, a highly valued edible and medicinal mushroom, is widely distributed in regions of South-Central China [2]. Researchers have identified various nutrients, such as polysaccharides, fatty acids, proteins, and amino acids [3], which contribute to its antioxidative, hypolipidemic [4], antidiabetic [5], and antiproliferative properties.

For most edible mushrooms, flavor is a critical factor influencing their market value and consumer preference [6]. Flavor is a multifaceted sensation that arises from the interaction of various compounds rather than being attributable to a single substance. The major compounds that determine flavor include amino acids, amino acid derivatives, and 5′-nucleotides [7]. Amino acids are categorized based on their taste profiles: aspartic acid (Asp) and glutamic acid (Glu) produce umami flavor; threonine (Thr), serine (Ser), glycine (Gly), and alanine (Ala) are responsible for sweetness; arginine (Arg), histidine (His), isoleucine (Ile), leucine (Leu), phenylalanine (Phe), methionine (Met), valine (Val), and tryptophan (Trp) generate a bitter taste; and cysteine (Cys), tyrosine (Tyr), and lysine (Lys) are relatively tasteless [8,9]. The composition of flavor compounds is influenced by the nature of the drying treatment adopted and the developmental stages, as demonstrated in species such as *Lentinula edodes* and *Dictyophora indusiata* [7,10,11]. Furthermore, different parts of the same mushroom species may exhibit distinct flavor profiles depending on the metabolic processes that are at play [12].

Polysaccharides, a major class of carbohydrates found in nature, are major components of edible mushrooms. Advances in science and technology have enabled the discovery of numerous polysaccharides in mushrooms and the characterization of their biological activities. For instance, *Phellinus linteus* polysaccharides (PL-N1) have been found to exert antitumor effects [13], *Hericium erinaceus* polysaccharides (EP-1) exhibit anti-chronic atrophic gastritis effects [14], and *Flammulina velutipes* polysaccharides (FVPB2) can improve immune function [15]. Evidence from studies exploring *L. decastes* polysaccharides supports their antitumor, anti-inflammatory, and antioxidant activities. Wang et al. [5] showed that *L. decastes* polysaccharides modulate gut microbiota to exert anti-obesity effects, and Zhang et al. [16] confirmed that these polysaccharides stimulate the Nrf2 signaling pathway, thereby ameliorating the degree of acute liver injury. While numerous studies have established that fungal polysaccharides are potent bioactive macromolecules with significant therapeutic effects against a wide range of diseases, the biosynthetic pathways and their dynamic alterations during fruiting body development remain largely unexamined. Investigating these polysaccharide biosynthetic routes in edible mushrooms is thus crucial for providing the foundational knowledge necessary for the rational engineering and large-scale production of these valuable biomolecules.

In this study, we investigated the dynamic changes in flavor and polysaccharides across various developmental stages of *L. decastes*. Metabolomic and transcriptomic analyses were conducted, which characterized the biosynthesis of flavor compounds and polysaccharides. The present findings provide important data that will guide the determination of the optimal harvesting stage of *L. decastes* to maximize its nutritional and flavor benefits, as well as expanding the current understanding of changes in flavor and polysaccharides, which is essential for the effective cultivation and utilization of this mushroom.

## 2. Materials and Methods

### 2.1. Strain and Culture Conditions

**Figure 1:**
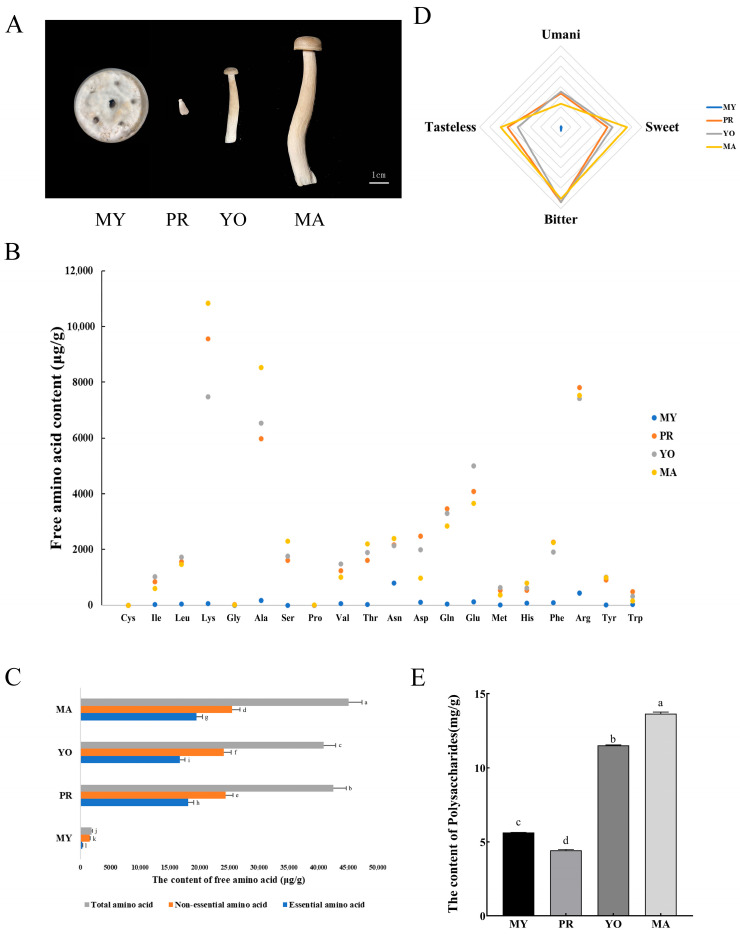
The concentrations of animo acids and polysaccharides at various developmental stages of *L. decastes*. (**A**) The developmental stages of *L. decastes*. Panels (**B**,**C**) present the concentrations of free amino acids, with panel (**B**) detailing the content of 20 specific amino acids and panel (**C**) showing the total amino acids, including both essential and non-essential types. (**D**) The flavor profiles of amino acids. (**E**) The content of polysaccharides. The stages are defined as follows: MY represents the mycelial stage, characterized by vegetative growth; PR denotes the primordial stage, marked by compact hyphal knots indicating the initial commitment to fruiting body formation; YO refers to the young fruiting body on the 16th day after substrate surface scratching, and MA represents the mature fruiting body ready for harvesting on the 25th day after substrate surface scratching. Different lowercase letters indicate statistically significant differences (*p* < 0.05).

The *L. decastes* strain was obtained from the Shandong Academy of Agricultural Sciences. It was cultivated under conditions adapted from a study by Liang et al. [17]. Initially, the fungus was cultured on potato dextrose agar at 25 °C for 7 days and then transferred to sterilized bottles containing a substrate mixture composed of 32% sawdust, 21% corn cob, 17% wheat bran, 15% corn flour, 7% soybean peel, 5% cottonseed hulls, and 3% soybean meal, calculated on a dry weight basis. Once the substrate was fully colonized by mycelia, the temperature of the cultivation environment was adjusted to 18 °C, and the surface of the substrate was lightly scratched to induce primordium formation. The bottle caps were then removed to allow for the development of basidiomata under a relative humidity of 95%. Samples were collected from various developmental stages, including the mycelial stage (MY), which represents the vegetative growth phase; the primordial stage (PR), which refers to the 11th day after substrate surface scratching and is characterized by compact hyphal knots that signify the initial commitment to fruiting body formation; and the fruiting body stages, which were further divided into the young fruiting body stage (YO) and the mature fruiting body stage (MA) for subsequent analysis (Figure 1A). The YO stage refers to the fruiting body on the 16th day after substrate surface scratching, during which structures are differentiated into a cap-and-stipe morphology, and the length of the fruiting body is 3–5 cm. In contrast, the MA stage denotes the mature fruiting bodies used for harvesting, which are collected on the 25th day after substrate surface scratching, and the length of the fruiting body is 11–13 cm.

### 2.2. Determination of Free Amino Acids

The concentration of free amino acids was determined using high-performance liquid chromatography (HPLC) (Thermo Scientific UltiMate 3000, Waltham, MA, USA). Briefly, the sample was prepared as follows: 100 mg of freeze-dried basidiomata was dispersed in 10 mL of pure water and ultrasonicated for 30 min. Subsequently, 1 mL of dichloromethane was added to the mixture, followed by centrifugation at 13,000 rpm for 5 min at 4 °C. The supernatant was collected and filtered through a 0.22 μm membrane. Finally, it was analyzed using HPLC on a C18-Hypersil Gold column (Thermo Scientific, 3 μm, 100 mm × 2.1 mm). The mobile phases consisted of 0.1% formic acid (*v*/*v*) as phase A and 100% methanol as phase B. The gradient elution was as follows: 0–0.5 min, 4% B; 0.5–2.5 min, linear increase to 35% B; 2.5–4.5 min, linear increase to 77% B; 4.5–6.5 min, linear increase to 100% B; 6.5–6.6 min, rapid decrease to 4% B; and 6.6–10 min, maintained at 4% B. The flow rate was set to 0.2 mL/min, with an injection volume of 3 μL and a column temperature of 40 °C. Mass spectrometry was performed with an electrospray ionization (ESI) source, ion spray voltage of 3500 V, sheath gas flow rate of 35 Arb, auxiliary gas flow rate of 10 Arb, and capillary temperature of 325 °C.

### 2.3. Determination of Polysaccharides

Next, 1 g of dried sample powder, dried to a constant weight and passed through a 40-mesh sieve, was mixed with 42 mL of ultrapure water in a 50 mL centrifuge tube. The mixture was heated at 65 °C for 2.5 h, cooled, and centrifuged at 7500 rpm for 10 min. The supernatant was collected and concentrated to 1/5 of its original volume by drying on a rotary evaporator at 55 °C. The concentrated supernatant was then cooled, and 3 times its volume of anhydrous ethanol was added, mixed, and allowed to stand for 24 h at 4 °C. After centrifugation at 7500 rpm for 10 min, the supernatant was discarded, and the precipitate was collected and dried at 60 °C in a blast drying oven. The dried precipitate was dissolved in distilled water and centrifuged at 7500 rpm for 10 min to collect the supernatant, which was mixed with twice its volume of Sevag reagent (chloroform: n-butanol = 4:1) and incubated at 45 °C for 30 min. It was subsequently centrifuged at 4000 rpm for 10 min to remove the protein precipitate. This step was repeated 2–3 times until no precipitate remained, yielding a protein-free solution. The protein-free sample solution was dialyzed in a 3500 Da dialysis bag with ultrapure water for 48 h, with the water changed every 12 h to eliminate low-molecular-weight impurities. Next, the solution was freeze-dried to obtain purified polysaccharide powder, which was dissolved in ultrapure water to prepare a 1 mg/mL solution. The solution was quantified by measuring the absorbance at 490 nm with a microplate reader, and the content of polysaccharides was estimated using a glucose standard curve. Varying concentrations of glucose were prepared, and color was developed using the phenol–sulfuric acid method. Finally, the absorbance was recorded to construct a standard curve.

### 2.4. Metabolomic

Samples collected from different growth stages were freeze-dried using a Scietx-100F vacuum freeze dryer and then ground using a mixer mill (MM 400, Retsch, Düsseldorf, Germany) equipped with a zirconia bead at 30 Hz for 1.5 min. Subsequently, 1.2 mL of 70% methanol was added to 100 mg of the lyophilized powder and vortexed intermittently for a total of six cycles, each lasting 30 s. The preparation was stored at 4 °C overnight. The samples were centrifuged at 12,000 rpm for 10 min, filtered through a 0.22 μm membrane, and then subjected to an ultra-performance liquid chromatography–tandem mass spectrometry (UPLC-MS/MS) analysis using an ExionLC™ AD (Framingham, MA, USA).

The UPLC-ESI-MS/MS system was utilized to extract and analyze the metabolites. Separation was performed on an Agilent SB-C18 column (1.8 µm, 2.1 mm × 100 mm). The mobile phase consisted of two solvents: pure water with 0.1% formic acid (solvent A) and acetonitrile with 0.1% formic acid (solvent B). The gradient elution program was as follows: an initial composition of 95% A and 5% B, followed by a linear gradient to 5% A and 95% B over nine minutes, and then maintained at 5% A and 95% B for one minute. The composition was rapidly adjusted back to 95% A and 5% B within 1.1 min and allowed to stand for an additional 2.9 min. The flow rate was maintained at 0.35 mL/min, with the column oven temperature set to 40 °C and an injection volume of 4 µL. The eluent was subjected to a QTRAP-MS system equipped with an ESI-triple quadrupole detector. The metabolite analysis protocol was adapted from a previously published study [18].

The metabolite data were statistically analyzed, with log2 transformation and normalization applied to enhance data normality. A hierarchical clustering analysis (HCA) and principal component analysis (PCA) were performed in R software 3.5.1 to characterize metabolite accumulation patterns across different accessions. Statistical significance was set to a *p*-value of 0.05 and a fold change of 2.0. Venn diagrams were constructed to illustrate the number of differentially accumulated metabolites. Furthermore, the Kyoto Encyclopedia of Genes and Genomes (KEGG) database was utilized to analyze differential metabolites, with a significance level of *p* < 0.01. The data were visualized using GraphPad Prism version 6.01 (GraphPad Software Inc., La Jolla, CA, USA).

### 2.5. Transcriptomic Analysis

Total RNA was isolated from samples at various growth stages using an RNAprep Pure Plant Kit (DP441, Tiangen, Beijing, China). The integrity and quality of the RNA were determined using an Agilent Bioanalyzer 2100 system (Agilent Technologies, Santa Clara, CA, USA) and a NanoPhotometer spectrophotometer (IMPLEN, Westlake Village, CA, USA). The mRNA was enriched with oligo (dT) magnetic beads and randomly fragmented. First-strand cDNA synthesis was performed using the M-MuLV reverse transcriptase system. Subsequently, second-strand cDNA was prepared using DNA polymerase after RNA degradation by RNase H. Adapters were ligated to the double-stranded cDNA for sequencing. This was followed by the construction of cDNA libraries, which were then amplified, purified, and sequenced on an Illumina Novaseq6000 platform in conjunction with AMPureXP beads (Beckman Coulter, Brea, CA, USA).

Original image data were transformed into sequence reads (raw reads) using CASAVA 2.0 base recognition software. The Fastp tool was employed to eliminate adapters and low-quality sequences containing more than 50% of bases with a Qphred score ≤ 20. Next, we calculated the GC content of the cleaned reads and applied FastQC to determine the Q20 and Q30 values to assess base quality. In addition, RNA-seq data were assembled and analyzed de novo if there was no reference genome. Gene expression levels were quantified using the Fragments per Kilobase of transcript per Million fragments mapped (FPKM) metric. Differentially expressed genes (DEGs) were identified based on the log2 fold change in expression in at least one direction, with a false discovery rate (FDR) ≤ 0.05. Gene functions were annotated based on the Nr, Pfam, Swiss-Prot, KEGG, and GO databases. The transcriptome raw data were uploaded to NCBI’s Sequence Read Archive with accession numbers SRR24926094-SRR24926096 for mycelia (55d 1–3); SRR24926090, SRR24926104, and SRR24926105 for primordia (PR 1–3); SRR24926101-SRR24926103 for young fruiting bodies (YO 1–3); and SRR24926098-SRR24926100 for mature fruiting bodies (MA 1–3).

### 2.6. Statistical Analysis

The presented data were obtained from a minimum of 3 independent sample measurements and are expressed as the mean ± standard error (SE). Error bars represent standard deviations calculated from the means of triplicate samples. All statistical analyses were performed using GraphPad Prism 6 (GraphPad Software Inc., La Jolla, CA, USA). Group comparisons were conducted using a one-way analysis of variance (ANOVA) followed by Tukey’s honest test. *p* < 0.05 was considered statistically significant.

## 3. Results

### 3.1. Free Amino Acids and Polysaccharides During Developmental Stages of L. decastes

*L. decastes* is highly favored by consumers for its unique flavor and nutritional value. To monitor the nutrition and bioactive components of *L. decastes* during the developmental process, the concentrations of free amino acids and polysaccharides in the mycelia, primordia, young fruiting bodies, and mature fruiting bodies were measured (Figure 1A).

Studies have shown that flavor-associated amino acids exist in a free state and are involved in the formation of flavor compounds [19], which can be directly absorbed and utilized by the body. The amino acids enriched at different developmental stages are shown in Figure 1B. It was observed that lysine was the most abundant free amino acid, with a concentration of 10,841.1 μg/g in the MA stage, followed by alanine, arginine, glutamic acid, and glutamine. In addition, the concentrations of total amino acids (45,107.39 μg/g), essential amino acids (19,553.85 μg/g), and non-essential amino acids (25,553.54 μg/g) in the MA stage were significantly higher than those in the other developmental stages (Figure 1C). Generally, amino acids are classified into four categories based on their flavor profiles [9] as follows: umami (Glu and Asp), sweetness (Ala, Thr, Ser, Gly, and Pro), bitterness (Arg, Phe, His, Val, Leu, Ile, and Met), and tasteless (Lys, Tyr, and Cys) [20]. Therefore, the determined amino acids were classified, and a radar chart analysis was conducted to elucidate the flavor changes of amino acids at different developmental stages of *L. decastes*. It was observed that sweet and tasteless amino acids were highest in the MA stage (estimated at 13,046.47 μg/g and 11,824.06 μg/g, respectively) compared to in the other stages (Figure 1D). Furthermore, the contents of umami and bitter amino acids were highest in the YO stage, estimated at 6982.74 μg/g and 14,811.39 μg/g, respectively.

Further analysis showed that polysaccharides varied significantly across different developmental stages (Figure 1E). The content of polysaccharides was highest in the MA stage (13.66 mg/g), followed by the YO (11.53 mg/g) and MY (5.63 mg/g) stages, with its content in the PR stage being the lowest (4.42 mg/g). Collectively, these results show that the total amino acids and polysaccharides were highest in the mature stage of *L. decastes*, while umami-associated amino acids were highest in the young fruiting body. To further characterize amino acid and polysaccharide biosynthesis at various developmental stages in *L. decastes*, metabolomic and transcriptomic analyses were performed to identify the key genes associated with the identified active metabolic processes.

### 3.2. Metabolomic Profiles During L. decastes Development

#### 3.2.1. PCA and Metabolite Composition Analysis

**Figure 2:**
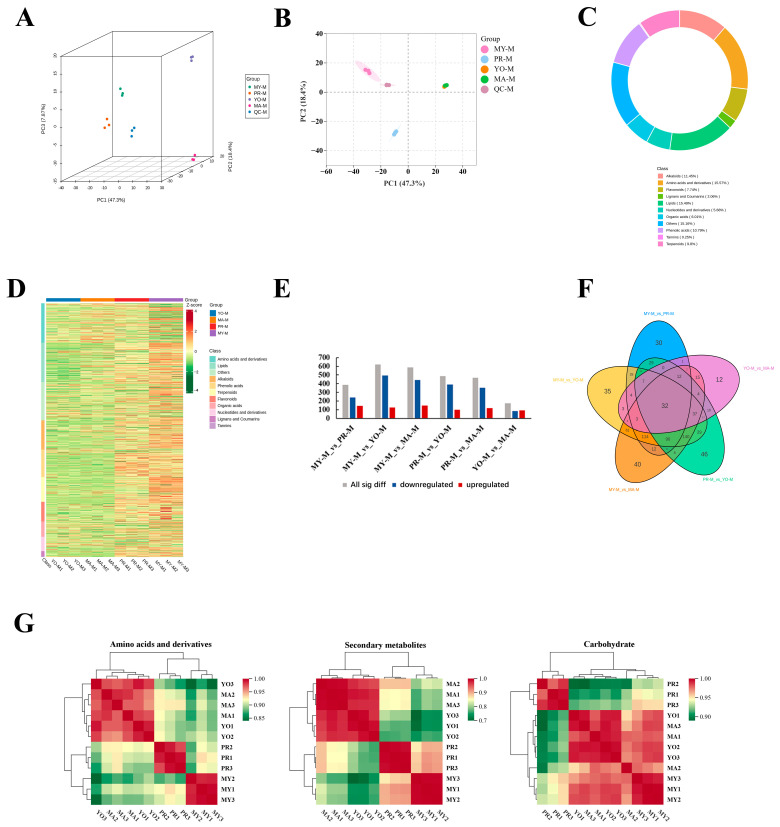
Metabolomic analysis of *L. decastes*. Panels (**A**,**B**) show three-dimensional and two-dimensional principal component analyses, respectively. (**C**) Analysis of metabolite class composition. (**D**) Heat map of all metabolites. (**E**) Differentially accumulated metabolites at different developmental stages. (**F**) Venn diagram analysis of differentially accumulated metabolites. (**G**) Correlation analysis of differential metabolites. MY, mycelia; PR, primordia; YO, young fruiting bodies collected on the 16th day after substrate surface scratching; MA, mature fruiting bodies ready for harvesting on the 25th day after substrate surface scratching.

To analyze and compare the differentially accumulated metabolites (DAMs) across different developmental stages, the UPLC-ESI-MS/MS system was employed. Three-dimensional principal component analysis (3D-PCA) score plots were constructed to demonstrate the differences among the developmental stages (Figure 2A). The total three-dimensional PCA accounted for 73.57% of the total variance (PC1 = 47.3%, PC2 = 18.4%, and PC3 = 7.87%), clearly distinguishing the different developmental stages. The score plot further indicated that the metabolic profiles of the two fruiting body developmental groups (YO and MA) exhibited greater similarity (Figure 2B). Although the differences were significant, they were mainly caused by the lower levels of most metabolites in the PR group. This indicates that the metabolites were altered by the developmental transition from vegetative to reproductive growth during primordia formation. Therefore, we analyzed the composition of metabolite classes, as shown in Figure 2C. Overall, the results revealed that the most significantly altered components in the four developmental stages were amino acids and their derivatives (15.57%). Moreover, secondary metabolites were altered throughout the developmental stages, including alkaloids (11.45%), terpenoids (9.8%), flavonoids (7.74%), and lignans and coumarins (2.06%). The identified metabolites are shown in a heat map presented in Figure 2D.

#### 3.2.2. Differential Metabolite Analysis

A pairwise comparison of the four developmental stages was performed to characterize the metabolites contributing to the differences in quality (Figure 2E). The results revealed significant differences in the DAMs between MY vs. PR, MY vs. YO, MY vs. MA, PR vs. YO, and PR vs. MA. This indicates that the most substantial metabolic changes occurred from the vegetative stage to the reproductive growth stages, with the PR vs. YO and PR vs. MA comparisons showing subsequent rearrangements. During the transition from the vegetative stage to reproductive growth, 385, 620, and 587 DAMs were detected in MY vs. PR, MY vs. YO, and MY vs. MA, respectively. Moreover, 488 and 469 DAMs were identified during fruiting body formation (PR vs. YO and PR vs. MA, respectively). However, only 173 DAMs were altered in the YO vs. MA comparison. The primordia represented the transition from vegetative growth to reproductive growth, characterized by morphological changes, as well as metabolite rearrangement.

In a Venn analysis, 32 metabolites (41% lipids and 22% secondary metabolites) were identified as key differential metabolites during the developmental stages (Figure 2F and Supplementary Material Table S1). Furthermore, 46, 40, 35, 30, and 12 specific metabolites were identified in the PR vs. YO, MY vs. MA, MY vs. YO, MY vs. PR, and YO vs. MA comparisons, respectively. To investigate the correlation between the differential metabolites and different developmental stages, a clustering analysis was conducted based on the quantitative data of the metabolites (Figure 2G). The correlation analysis revealed that the accumulation patterns of the metabolites involved in amino acid and secondary metabolite biosynthesis were similar. Primarily, amino acids and secondary metabolites were closely associated with the YO and MA stages. However, carbohydrates were common among the MY, YO, and MA stages.

### 3.3. Transcriptomic Profiles During L. decastes Development

**Figure 3:**
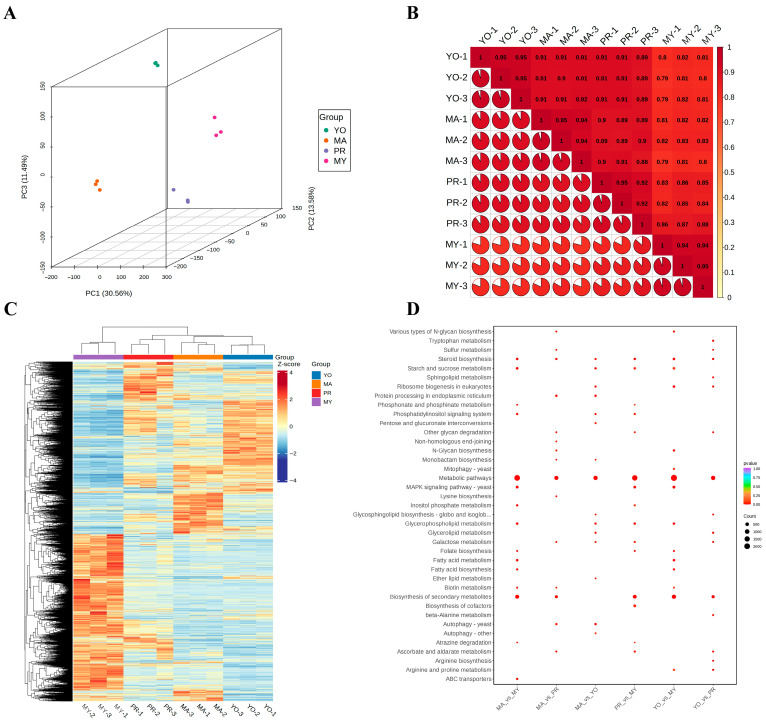
Transcriptomic analysis of *L. decastes*. (**A**) Three-dimensional principal component analysis (3D-PCA). (**B**) Pearson correlation coefficient. (**C**) Heatmap of all DEGs. (**D**) KEGG pathway enrichment analysis. MY, mycelia; PR, primordia; YO, young fruiting bodies collected on the 16th day after substrate surface scratching; MA, mature fruiting bodies ready for harvesting on the 25th day after substrate surface scratching.

To characterize the biosynthesis of nutritional ingredients in *L. decastes*, RNA sequencing was carried out using samples from different developmental stages of *L. decastes*. The analysis revealed significant differences across the developmental stages, as shown in a 3D-PCA score plot (Figure 3A). The PCA analysis showed clear clustering of the four developmental stages, with variances of PC1 = 30.56%, PC2 = 13.58%, and PC3 = 11.49%. The reproducibility of sample collection was assessed using Pearson’s correlation coefficient (Figure 3B). The results demonstrated high repeatability and strong correlations among samples from the same developmental stage. Notably, the correlation among samples in the MY stage was weaker than that in the other developmental stages, as observed through the analysis of repeatability across different stages. Significant correlations were found among PR, YO, and MA, with the results of the hierarchical heatmap clustering analysis showing significantly different profiles of the DEGs in MY compared with in PR, YO, and MA (Figure 3C). Most of the identified DEGs were highly expressed in MY gradually, but their expression decreased during primordia formation. Similarly, the DEGs that exhibited a low expression in MY showed an increased expression in PR, YO, and MA. Of note, the expression levels of the DEGs in YO and MA were similar during the primordial stage. The expression levels of the DEGs in PR fell within the range between those in MY and YO. These results show that the DEGs in PR are important modulators of the life cycle in *L. decastes*.

To further analyze the biological function of the DEGs, a KEGG pathway enrichment analysis was performed for various developmental stages (Figure 3D). It was observed that the DEGs were significantly enriched in the metabolic pathways of six groups, followed by the biosynthesis of secondary metabolites. Moreover, the DEGs were involved in the biosynthesis of secondary metabolites, including in MA vs. MY (714 DEGs), MA vs. PR (304 DEGs), PR vs. MY (626 DEGs), YO vs. MY (870 DEGs), and YO vs. PR (329 DEGs), except for MA vs. YO. These findings imply that secondary metabolites play crucial roles during the developmental cycle of *L. decastes*.

Furthermore, some DEGs were enriched in amino acid metabolism, including tryptophan metabolism, lysine biosynthesis, beta-alanine metabolism, arginine biosynthesis, and arginine and proline metabolism. Interestingly, the patterns of amino acid biosynthesis and metabolism varied across the different developmental stages. For instance, tryptophan metabolism (49 DEGs) and arginine biosynthesis (21 DEGs) were detected only in the YO vs. PR comparison, lysine biosynthesis (14 DEGs) was found only in the MA vs. PR comparison, and arginine and proline metabolism was detected in the YO vs. MY (93 DEGs) and YO vs. PR (54 DEGs) groups. These results imply that specific amino acid biosynthesis and metabolism are involved during the different developmental stages of *L. decastes*.

Moreover, several DEGs were enriched in carbohydrate metabolism pathways, including starch and sucrose metabolism, pentose and glucuronate interconversions, inositol phosphate metabolism, galactose metabolism, and ascorbate and aldarate metabolism. For instance, 25 DEGs (MA vs. PR), 28 DEGs (MA vs. YO), 48 DEGs (PR vs. MY), and 29 DEGs (YO vs. PR) were involved in galactose metabolism pathways. Therefore, we postulated that these DEGs might be involved in polysaccharide biosynthesis during the development of *L. decastes*.

### 3.4. Integrated Metabolomic and Transcriptomic Analyses

#### 3.4.1. KEGG Pathway

**Figure 4:**
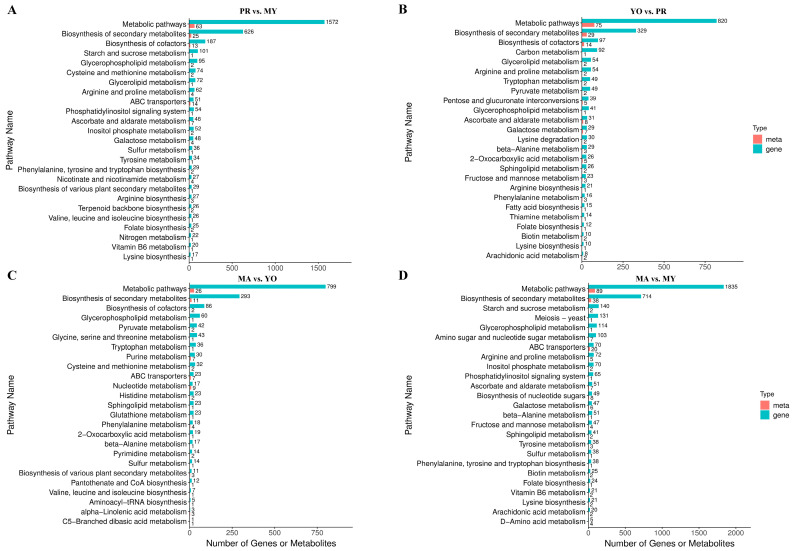
KEGG enrichment analysis. (**A**) PR vs. MY. (**B**) YO vs. PR. (**C**) MA vs. YO. (**D**) MA vs. MY. MY, mycelia; PR, primordia; YO, young fruiting bodies collected on the 16th day after substrate surface scratching; MA, mature fruiting bodies ready for harvesting on the 25th day after substrate surface scratching.

A KEGG pathway enrichment analysis was performed for the DEGs and DAMs to clarify their variations during the life cycle of *L. decastes*. The results indicate that the metabolomic data and transcriptomic results were largely consistent (Figure 4A–D). Specifically, DEGs and DAMs were enriched in various metabolic pathways and in the biosynthesis of secondary metabolites. The results revealed that the highest numbers of DEGs and DAMs were observed in the MA vs. MY comparison, suggesting the most significant differences between the MY and MA stages. In contrast, the MA vs. YO comparison exhibited the fewest DEGs and DAMs, indicating a smaller degree of variation between these stages.

Furthermore, the DEGs and DAMs were enriched in amino acid biosynthesis and metabolism pathways in the PR vs. MY comparison, including ‘cysteine and methionine metabolism’, ‘arginine and proline metabolism’, ‘tyrosine metabolism’, ‘phenylalanine, tyrosine and tryptophan biosynthesis’, ‘arginine biosynthesis’, ‘valine, leucine and isoleucine biosynthesis’, and ‘lysine biosynthesis’ (Figure 4A). In the YO vs. PR comparison, the DEGs and DAMs were enriched in ‘arginine and proline metabolism’, ‘tryptophan metabolism’, ‘lysine degradation’, ‘bata-alanine metabolism’, ‘arginine biosynthesis’, ‘phenylalanine metabolism’, and ‘lysine biosynthesis’ (Figure 4B). In the MA vs. YO comparison, they were enriched in ‘glycine, serine and threonine metabolism’, ‘tryptophan metabolism’, ‘cysteine and methionine metabolism’, ‘histidine metabolism’, ‘phenylalanine metabolism’, ‘beta-alanine metabolism’, and ‘valine, leucine and isoleucine biosynthesis’ (Figure 4C). In the MA vs. MY comparison, they were enriched in ‘arginine and proline metabolism’, ‘beta-alanine metabolism’, ‘tyrosine metabolism’, ‘phenylalanine, tyrosine and tryptophan biosynthesis’, ‘lysine biosynthesis’, and ‘D-amino acid metabolism’ (Figure 4D). Notably, the results infer that the ‘lysine biosynthesis’ pathway may be highly active, as it was present in three of the four comparison groups: PR vs. MY (17 DEGs and 1 DAM), YO vs. PR (10 DEGs and 1 DAM), and MA vs. MY (21 DEGs and 2 DAMs). This suggests that lysine biosynthesis may contribute to the high abundance of lysine compared to all other free amino acids (Figure 1B).

A total of 197 DEGs and 12 DAMs were identified as being involved in carbohydrate metabolism, including ‘starch and sucrose metabolism’, ‘ascorbate and aldarate metabolism’, and ‘galactose metabolism’ in the PR vs. MY comparison. Furthermore, 171 DEGs and 25 DAMs involved in carbohydrate metabolism were identified in the YO vs. PR comparison, including ‘pyruvate metabolism’, ‘pentose and glucuronate interconversions’, ‘ascorbate and aldarate metabolism’, ‘galactose metabolism’, and ‘fructose and mannose metabolism’. We further identified 42 DEGs and 2 DAMs in the MA vs. YO comparison, including ‘pyruvate metabolism’. Moreover, 355 DEGs and 21 DAMs were detected in the MA vs. MY comparison, including ‘starch and sucrose metabolism’, ‘inositol phosphate metabolism’, ‘ascorbate and aldarate metabolism’, ‘galactose metabolism’, and ‘fructose and mannose metabolism’. The results show that the highest numbers of DEGs and DAMs associated with carbohydrate metabolism were detected in the MA vs. MY comparison, while the MA vs. YO comparison exhibited the fewest. Notably, although the number of DEGs was higher in PR vs. MY than in YO vs. PR, the number of DAMs was significantly greater in YO vs. PR. This suggests that carbohydrate production may be most active during the fruiting body formation stages, particularly in PR, YO, and MA.

Collectively, these data indicate that most DEGs and DAMs are associated with metabolism, mainly secondary metabolite, amino acid, and carbohydrate metabolism, during the development of *L. decastes*.

#### 3.4.2. Biosynthesis of Various Secondary Metabolites

**Figure 5:**
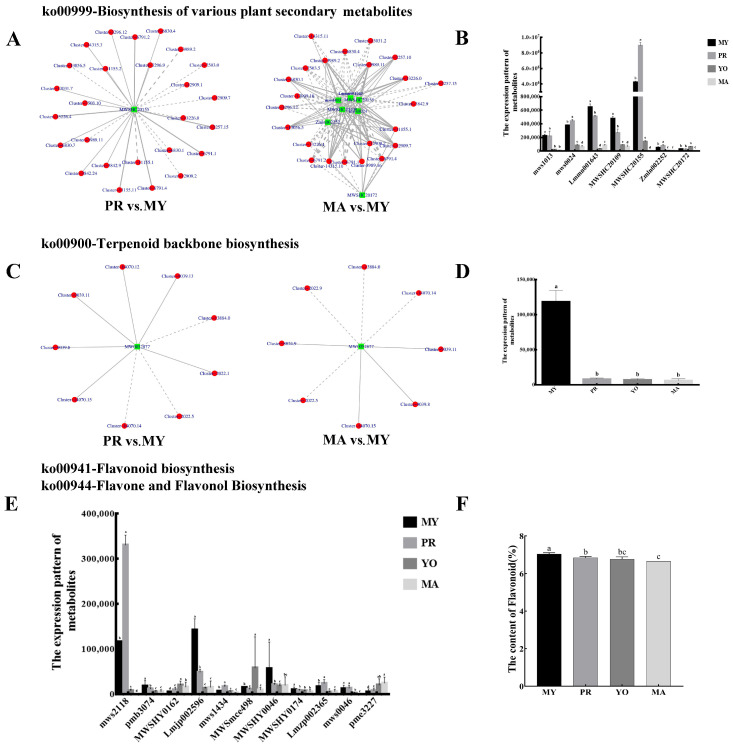
Secondary metabolite biosynthesis during the development of *L. decastes*. (**A**,**C**) Pearson correlation analysis between metabolites and genes. (**B**,**D**,**E**) Expression patterns of metabolites. (**F**) Total flavonoid content. (**A**,**B**) Biosynthesis of various plant secondary metabolites. (**C**,**D**) Terpenoid backbone biosynthesis. (**E**) Flavonoid biosynthesis and flavone and flavonol biosynthesis. The green box represents a metabolite, and the red circle represents a gene. The solid line represents a positive correlation, and the dashed line represents a negative correlation. MY, mycelia; PR, primordia; YO, young fruiting bodies collected on the 16th day after substrate surface scratching; MA, mature fruiting bodies ready for harvesting on the 25th day after substrate surface scratching. Different lowercase letters indicate significant differences (*p* < 0.05).

To characterize secondary metabolite biosynthesis during the development of *L. decastes*, an association analysis between metabolites and gene expression was conducted. The results show that one metabolite (Scopoletin, MWSHC20155) and seven metabolites, namely, Scopoletin (MWSHC20155), Esculetin (mws1013), Gallic acid (mws0024), 2-Hydroxycinnamic acid (Lmmn001643), coumarin (MWSHC20109), Sideretin (Zmln002252), and Isoscopoletin (MWSHC20172), were associated with the biosynthesis pathway in the PR vs. MY and MA vs. MY comparisons, respectively (Figure 5A). One of the identified metabolites (Scopoletin) was common to both groups and had the highest abundance among all metabolites involved in this pathway. Notably, its expression in the primordial stage was significantly higher than that in the mycelial and mature stages (Figure 5B). Furthermore, 26 DEGs (10 negatively regulated genes and 16 positively regulated genes) and 23 DEGs (12 negatively regulated genes and 11 positively regulated genes) were involved in the regulation of the Scopoletin metabolite in the PR vs. MY and MA vs. MY comparisons, respectively. In addition, 16 DEGs were common in the two groups (PR vs. MY and MA vs. MY), and their expression patterns were opposite.

Terpenoid is an important secondary metabolite in fungi. In this study, one metabolite (2-trans,6-trans-Farnesal, MW0052877) was identified during terpenoid backbone biosynthesis (Figure 5C). Moreover, nine DEGs (three negatively regulated genes and six positively regulated genes) and eight DEGs (four negatively regulated genes and four positively regulated genes) were identified as regulators of terpenoid backbone biosynthesis in the PR vs. MY and MA vs. MY comparisons, respectively. Six DEGs (*Cluster-13884.0*, *Cluster-2022.5*, *Cluster-14070.14*, *Cluster-14070.15*, *Cluster-9039.8*, and *Cluster-9039.11*) regulated common genes in both the PR vs. MY and MA vs. MY groups, with similar regulatory modes. The results also show that the content of 2-trans,6-trans-Farnesal (MW0052877) decreased during the growth of *L. decastes*, with the highest level observed in the mycelial stage (Figure 5D).

Recent studies have demonstrated that fungi contain high amounts of flavonoids [21]. In this study, eleven metabolites involved in flavonoid biosynthesis were identified through a metabolomic analysis (Figure 5E). Most metabolites were highly expressed in the mycelial or primordial stages. In addition, the expression levels of four metabolites, namely, 5-O-p-Coumaroylquinic acid (pmb3074), Quercetin-3-O-sambubioside (Lmjp002596), Quercetin-3-O-glucoside (MWSHY0046), and Epicatechin (MWSHY0174), were highest in MY. In comparison, the abundance of four metabolites, namely, Phlorizin (mws2118), Apigenin-6-C-glucoside (mws1434), Hesperetin-7-O-glucoside (Lmzp002365), and Naringin (mws0046), were highest in PR. Moreover, the total flavonoid content was measured during different developmental stages (Figure 5F). The results show that the total flavonoid content was highest in the mycelia, followed by the primordia and young fruiting body. However, the flavonoid content was lowest in the mature fruiting body. Notably, the experimental results were consistent with the findings from the metabolomic data.

#### 3.4.3. The Biosynthesis of Amino Acids

A Pearson correlation analysis was conducted to explore the association of the amino acids with the growth and development of *L. decastes*. Four pathways involved in the biosynthesis of amino acids were identified.

**Figure 6:**
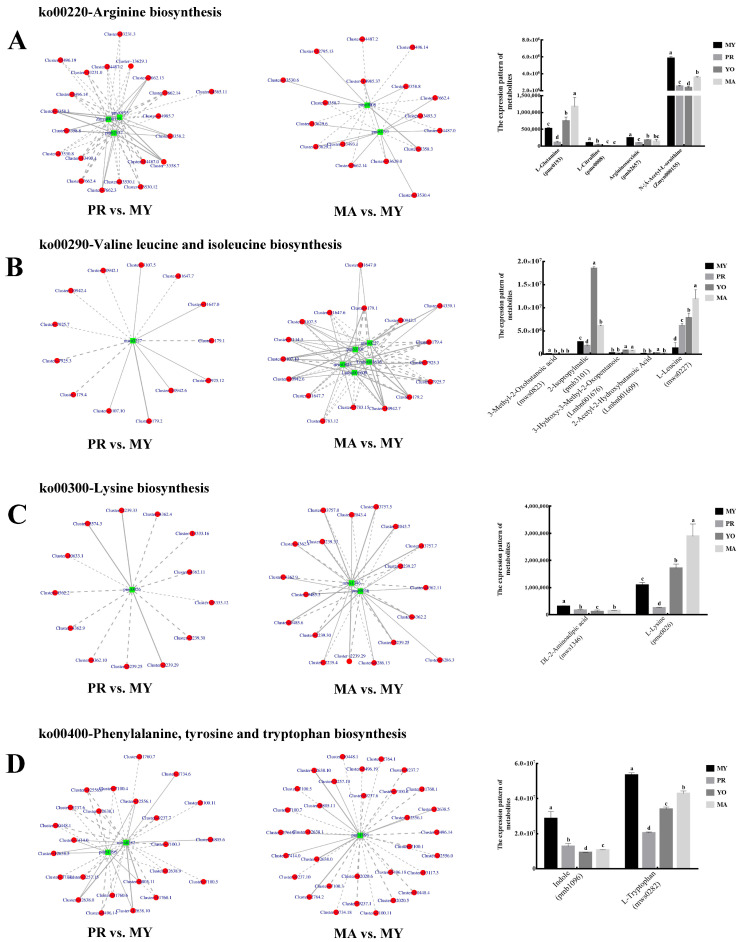
Amino acid biosynthesis in *L. decastes* development. (**A**) Arginine biosynthesis pathway. (**B**) Valine, leucine, and isoleucine biosynthesis pathway. (**C**) Lysine biosynthesis pathway. (**D**) Phenylalanine, tyrosine, and tryptophan biosynthesis pathway. The green box represents a metabolite, and the red circle represents a gene; solid lines represent positive corrections and dashed lines indicate negative corrections. MY, mycelia; PR, primordia; YO, young fruiting bodies collected on the 16th day after substrate surface scratching; MA, mature fruiting bodies ready for harvesting on the 25th day after substrate surface scratching. Different lowercase letters represent significant differences (*p* < 0.05).

In the arginine biosynthesis pathway, three DAMs were identified in the PR vs. MY comparison, while two were detected in the MA vs. MY comparison. Among them, L-Glutamine (pme0193) was a common metabolite present in both groups. Moreover, in the PR vs. MY comparison, 21 DEGs were involved in the regulation of Argininosuccinic acid (pmb2657), N-α-Acetyl-L-ornithine (Zmyn000155), and L-Glutamine (pme0193) (Figure 6A). Six DEGs (*Cluster-7662.13*, *Cluster-3358.2*, *Cluster-3358.7*, *Cluster-7662.3*, *Cluster-3358.8*, and *Cluster-3358.3*) were positively correlated with these three DAMs, while the remaining DEGs were negatively correlated. Furthermore, two DAMs were identified in the MA vs. MY comparison, namely, L-Citrulline (pme0008) and L-Glutamine, and L-Glutamine was also detected in the PR vs. MY analysis. Nine DEGs (*Cluster-14487.2*, *Cluster-5496.14*, *Cluster-3358.8*, *Cluster-7662.4*, *Cluster-14487.0*, *Cluster-3358.3*, *Cluster-7662.14*, *Cluster-13493.1*, and *Cluster-3358.7*) were predicted to regulate the accumulation of metabolites in both the PR vs. MY and MA vs. MY groups.

An analysis of the valine, leucine, and isoleucine biosynthesis pathway revealed that L-Leucine (mws0227) was a common metabolite in the two groups, PR vs. MY and MA vs. MY (Figure 6B). L-Leucine (mws0227) was regulated by 13 DEGs, 6 of which were positively correlated in PR vs. MY. In the MA vs. MY comparison, four additional DAMs were identified: 3-methyl-2-oxobutanoic acid (mws0823), 2-isopropylmalic acid (pmb3101), 3-hydroxy-3-methyl-2-oxopentanoic acid (Lmbn001676), and 2-acetyl-2-hydroxybutanoic acid (Lmbn001609). Eleven DEGs (*Cluster-11647.0*, *Cluster-179.1*, *Cluster-10942.1*, *Cluster-179.4*, *Cluster-7925.3*, *Cluster-7925.7*, *Cluster-179.2*, *Cluster-11647.7*, *Cluster-10942.6*, *Cluster-3107.10*, and *Cluster-3107.5*) were predicted to be important regulators of the metabolites in the two comparisons, PR vs. MY and MA vs. MY.

In the lysine biosynthesis pathway, one common metabolite, L-Lysine (pme0026), was identified in the two comparisons: PR vs. MY and MA vs. MY (Figure 6C). Furthermore, another metabolite, DL-2-aminoadipic acid (mws1346), was found in the MA vs. MY group comparison. Eight DEGs that could regulate the metabolites in the comparisons of PR vs. MY and MA vs. MY were identified, namely, *Cluster-2239.33*, *Cluster-4362.4*, *Cluster-4362.11*, *Cluster-2239.30*, *Cluster-2239.29*, *Cluster-2239.25*, *Cluster-4362.9*, and *Cluster-4362.2*.

In the phenylalanine, tyrosine, and tryptophan biosynthesis pathway, two DAMs, indole (pmb1096) and L-Tryptophan (mws0282), were identified in PR vs. MY (Figure 6D). One metabolite (indole) was detected in MA vs. MY, and it was also present in PR vs. MY. Sixteen DEGs were common among the significantly regulated genes in PR vs. MY and MA vs. MY. These included *Cluster-12556.0*, *Cluster-9237.6*, *Cluster-12638.1*, *Cluster-12556.1*, *Cluster-9237.7*, *Cluster-7100.11*, *Cluster-10448.1*, *Cluster-7414.0*, *Cluster-12638.5*, *Cluster-7100.3*, *Cluster-6805.11*, *Cluster-7100.5*, *Cluster-12638.0*, *Cluster-11760.1*, *Cluster-5496.14*, and *Cluster-12638.10*.

#### 3.4.4. Biosynthesis of Polysaccharides

**Figure 7:**
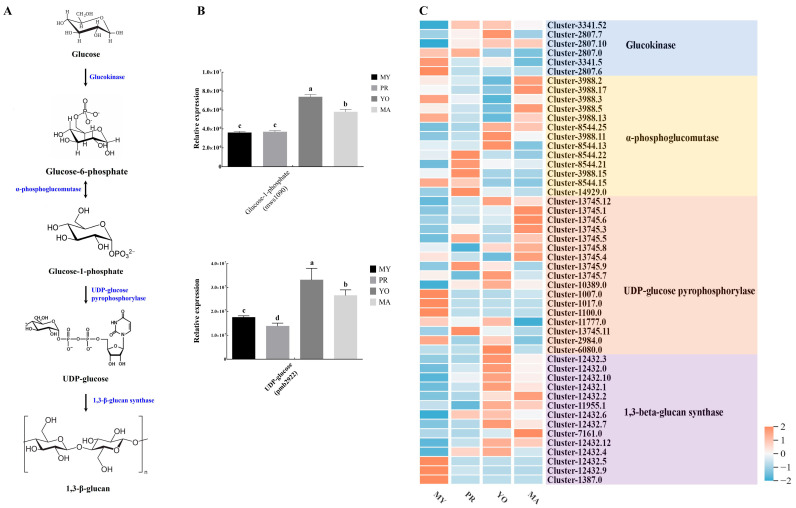
Polysaccharide biosynthesis during *L. decastes* development. (**A**) The polysaccharide biosynthesis pathway. (**B**) The relative expression levels of metabolites. (**C**) A heat map of key enzyme gene expression. MY, mycelia; PR, primordia; YO, young fruiting bodies collected on the 16th day after substrate surface scratching; MA, mature fruiting bodies ready for harvesting on the 25th day after substrate surface scratching. Different lowercase letters represent significant differences (*p* < 0.05).

The activity of *L. decastes* was mainly attributed to its polysaccharides, which have been shown to prevent the development of obesity, suppress immune activity [22], and modulate intestinal microflora [23]. Moreover, glucose, as a major component of polysaccharides that provides the carbon backbone for biosynthesis, was the most abundant. To understand the biosynthesis process of polysaccharides in *L. decastes*, we characterized the intermediate metabolites and genes encoding key enzymes (glucokinase, α-phosphoglucomutase, UDP-glucose pyrophosphorylase, and 1,3-β-glucan synthase) during various developmental stages (Figure 7A). Two intermediate metabolites, Glucose-1-phosphate (mws1090) and UDP-glucose (pmb2922), exhibited similar expression patterns, with their expression being highest in the YO stage, followed by the MA stage (Figure 7B). These components provided primary precursors for the biosynthesis of polysaccharides in the mature stage of *L. decastes*. These observations expand the current understanding of the polymerization process.

Furthermore, bioinformatic annotation identified 50 genes encoding putative enzymes, including glucokinase, α-phosphoglucomutase, UDP-glucose pyrophosphorylase, and 1,3-beta-glucan synthase (Figure 7C). Among the six genes encoding glucokinase, *Cluster-3341.52* exhibited the highest expression (Supplementary Material Table S2). The expression of this gene increased during the development of the fruiting body and decreased in the MA stage. The expression levels of 5 genes (*Cluster-3988.2*, *Cluster-3988.17*, *Cluster-3988.3*, *Cluster-3988.5*, and *Cluster-3988.13*) were similar and elevated among the 13 genes encoding α-phosphoglucomutase. Further analysis revealed that the expression of these genes decreased during fruiting body formation and increased in the MA stage. However, the other three genes (*Cluster-8544.25*, *Cluster-3988.15*, and *Cluster-8544.13*) encoding α-phosphoglucomutase exhibited opposite expression patterns. In addition, the expression levels of five genes encoding UDP-glucose pyrophosphorylase were significantly higher than those of the other genes, with three genes (*Cluster-13745.12*, *Cluster-13745.1*, and *Cluster-13745.6*) showing a progressive increase during the development of the fruiting body, whereas the expression level of *Cluster-13745.12* slightly decreased in the MA stage. The results show that the expression levels of *Cluster-13745.3* and *Cluster-13745.5* decreased in the YO stage, whereas the four genes encoding 1,3-beta-glucan synthase showed similar expression patterns, with their expression being highest in the YO stage but subsequently slightly decreased in the MA stage. Collectively, these results indicate that the abundance of metabolites and key enzyme genes associated with polysaccharide biosynthesis varied across the developmental stages.

### 3.5. The Key Metabolic Pathways of Nutrients and Flavor During the Development of L. decastes

**Figure 8:**
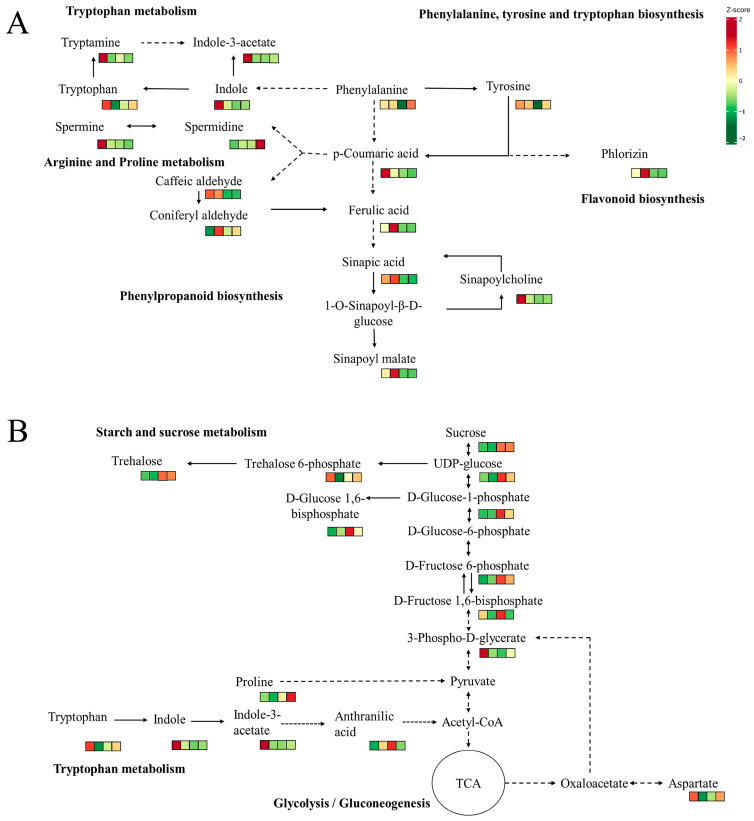
The key pathways for the formation of nutrients and flavor substances. (**A**) Phenylpropanoid biosynthesis and amino acid metabolic pathways. (**B**) Carbohydrate metabolism and amino acid metabolism. The solid line indicates direct generation, while the dashed line indicates that substances are generated through multiple reactions. The arrow indicates the direction of the reaction. These small squares, from left to right, represent the following: MY (mycelia); PR (primordia); YO (young fruiting bodies collected on the 16th day after substrate surface scratching); MA (mature fruiting bodies ready for harvesting on the 25th day after substrate surface scratching).

We propose that phenylpropanoid biosynthesis is interconnected with both amino acid and flavonoid metabolism (Figure 8A). Previous studies suggest that phenylalanine and tyrosine likely serve as precursors to *p*-coumaric acid, which may subsequently be integrated into phenylpropanoid, flavonoid, arginine, and proline pathways. The increased concentrations of *p*-coumaric acid and its potential downstream metabolites—phlorizin and spermine—observed during the MY and YO stages further substantiate this association. Similarly, phenylalanine-derived indole appears to lead to the synthesis of tryptophan, which is then converted to tryptamine, ultimately forming indole-3-acetate, all of which are most prevalent in the MY stage. Indole may also serve as a direct precursor for indole-3-acetate production.

Carbohydrate metabolism supports growth, development, and bacterial resistance in fruiting bodies by supplying essential nutrients. Sucrose produces UDP-glucose, which is converted to trehalose for osmotic balance and signaling, especially in the YO and MA stages (Figure 8B). UDP-glucose can also be transformed into pyruvate through glycolysis, which is then converted to acetyl-CoA for the TCA cycle, releasing energy. Tryptophan and proline are precursors of acetyl-CoA and pyruvate, while aspartate forms oxaloacetate, influencing the speed of the TCA cycle. These processes involve tryptophan metabolism, as well as arginine and proline metabolism.

The varying expression levels of metabolites in different stages indicate that the nutrients and flavor substances in the fruiting bodies undergo a series of complex changes. These changes reveal distinct nutritional profiles and flavor characteristics at each stage of *L. decastes* development.

## 4. Conclusions and Discussion

The developmental processes involved in the fruiting bodies of fungi are complex and highly regulated. The processes involve multiple stages, ranging from spore germination to the maturation of the fruiting body. This developmental process not only influences the reproductive success of the fungus but also determines the concentration of various nutritional components, such as amino acids and polysaccharides. Thus, it is imperative to investigate the interplay between fruiting body development and nutrient content to provide insights for optimizing the nutritional and medicinal value of mushrooms.

Our study, along with several others, demonstrates that the concentrations of various nutritional components in fungi vary across different developmental stages, such as in *Ganoderma lucidum* [24], *Lentinula edodes* [25], and *Inonotus hispidus* [26]. For instance, the major metabolites detected during the early stages of mycelial growth were simple sugars and amino acids [17,27], which are essential for the structural and metabolic needs of fungi. As the mycelium transitions, resulting in primordia formation and subsequent fruiting body development, the synthesis and accumulation of bioactive compounds, such as secondary metabolites and polysaccharides, are significantly enhanced. The present results demonstrate a significant increase in the amino acid and polysaccharide contents in the fruiting bodies of *L. decastes* as they mature, with the highest concentrations of these compounds detected in the mature stage. Thus, the mature stage of this mushroom may represent the ideal time for harvest to achieve optimal nutritional and medicinal value.

Mushrooms are abundant in various flavor compounds, with glutamic acid and aspartic acid primarily contributing to their umami taste. Notably, glutamic acid is present at the highest concentration, imparting a pronounced umami flavor to species such as *L. edodes*, *Pleurotus ostreatus*, and *Agaricus bisporus* [28]. However, our research indicates that bitterness is the most prominent flavor in *L. decastes*, with lysine emerging as the most prevalent free amino acid and glutamic acid ranking fourth in concentration. Furthermore, the levels of umami amino acids exhibit an initial increase followed by a decrease during the formation and development of fruiting bodies, peaking during the YO period. This observation diverges from that in previous studies. These findings may provide valuable insights into the optimal consumption period for *L. decastes*, thereby influencing the timing of harvest.

Research has shown that secondary metabolism in fungi is highly regulated during fungal growth and development [29]. Secondary metabolites were found to be significantly elevated as development progressed. Ren et al. [30] reported that secondary metabolites, such as total flavonoids and ganoderic acids A and D, were most abundant during the maturity stage of *G. lucidum* relative to the formative growth stages. In addition, the total flavonoid concentration was observed to progressively increase as the fruiting body of *Stropharia rugosoannulata* continued to grow [31]. However, the concentration of secondary metabolites in the mycelial stage was significantly higher than that in the developmental stages of the fruiting body of *L. decastes*. Our finding differs from that reported in a previous study.

The formation of fruiting bodies requires the synthesis of numerous proteins needed to aid differentiation into the various necessary cell types [32]. During this process, amino acids are used as precursors to mediate translation. In this study, metabolomic and transcriptomic analyses allowed us to understand the biosynthetic pathways contributing to the accumulation of amino acids and polysaccharides. Our analyses uncovered a significant rearrangement of metabolites and the differential expression of genes associated with secondary metabolite, amino acid, and carbohydrate metabolism, highlighting the dynamic nature of *L. decastes* development. These dynamic changes during development not only demonstrate its potential value in the fields of functional foods and nutritional supplements but may also guide the future utilization and cultivation of *L. decastes*.

Throughout the ontogeny of *L. decastes*, the development of the fruiting body is characterized by a meticulously orchestrated shift in both primary and secondary metabolism. By integrating metabolomic and transcriptomic analyses, we demonstrate that the transition from vegetative mycelium to mature basidiocarp is characterized by (i) a significant accumulation of amino acids, particularly lysine, and polysaccharides, which peak at maturity; (ii) a transient increase in umami-related amino acids, such as glutamate and aspartate, during the early fruiting stage; and (iii) a developmental re-prioritization of secondary metabolite gene expression that partially diverges from patterns observed in other mushroom species. These stage-specific metabolic signatures not only elucidate the optimal harvesting period for maximizing nutritional and medicinal value but also provide a molecular framework for future strain improvement and industrial cultivation strategies.

## Data Availability

The raw data supporting the conclusions of this article will be made available by the authors on request.

## Supplementary Materials

The following supporting information can be downloaded at: https://www.mdpi.com/article/10.3390/jof11080586/s1, Table S1. Venn diagram of *L. decastes* among different developmental stages. Table S2. The genes encoding metabolites involved in polysaccharide biosynthesis.
